# Cadherin 13 Inhibits Pancreatic Cancer Progression and Epithelial-mesenchymal Transition by Wnt/β-Catenin Signaling

**DOI:** 10.7150/jca.37762

**Published:** 2020-02-03

**Authors:** Dengfei Xu, Hui Yuan, Zihong Meng, Chunmei Yang, Zefang Li, Mengge Li, Zhigang Zhang, Yu Gan, Hong Tu

**Affiliations:** 1 State Key Laboratory of Oncogenes and Related Genes, Shanghai Cancer Institute, Renji Hospital, Shanghai Jiao Tong University School of Medicine, Shanghai 200032, China.; 2 Department of Thoracic Surgery, Cancer Research Center, Fudan University Shanghai Cancer Center, Shanghai 200032, China.; 3 Department of Orthopaedic Surgery, Zhongshan Hospital, Fudan University, Shanghai 200032, China.

**Keywords:** CDH13, pancreatic cancer, metastasis, epithelial-mesenchymal transition.

## Abstract

Cadherin 13 (CDH13) is an atypical cadherin that exerts tumor-suppressive effects on cancers derived from epithelial cells. Although the CDH13 promoter is frequently hypermethylated in pancreatic cancer (PC), the direct impact of CDH13 on PC is unknown. Accordingly, the expression of CDH13 in PC cell lines and paired PC tissues was examined by immunohistochemistry, quantitative real-time PCR and western blotting. Our findings showed that CDH13 was downregulated in PC tissues and cell lines. Moreover, cell proliferation, migration and invasion were detected by CCK-8 assay, transwell migration assay and transwell invasion assay, respectively. Xenograft tumor experiments were used to determine the biological function of CDH13 *in vivo*. As revealed by our data, CDH13 overexpression significantly inhibited the proliferation, migration and invasion of human PC cells *in vitro*. The inhibitory effect of CDH13 on PC was further confirmed in animal models. Mice subcutaneously or orthotopically transplanted with CDH13-overexpressing CFPAC-1 cells developed significantly smaller tumors with less liver metastases and mesenteric metastases than those of the control group. Next, transcriptomics and western blot analysis were used to identify the underlying mechanisms. Further molecular mechanism studies showed that CDH13 overexpression inhibited the activation of the Wnt/β-catenin signaling pathway and regulated the expression of epithelial-mesenchymal transition (EMT)-related markers. Our results indicated that CDH13 displayed an inhibitory effect on PC and suggested that CDH13 might be a potential biomarker and a new therapeutic target for PC.

## Introduction

Pancreatic cancer (PC) is one of the most dismal human malignancies with a steadily increasing incidence and a five-year survival rate of 7% 1. This low survival rate is attributed in part to the fact that PC metastasis often progresses to the point where surgical removal cannot provide a cure 2. Little improvement regarding the survival of PC patients has been achieved over the past 20 years 3. Therefore, it is crucial to discover novel potential biomarkers for early diagnosis and prognosis prediction and therapeutic targets for PC. Furthermore, the exploration and understanding of the molecular mechanisms that lead to metastatic dissemination in PC are necessary.

Cadherin 13 (CDH13, also known as T-cadherin or H-cadherin) is an atypical glycosylphosphatidylinositol-anchored member of the cadherin superfamily that lacks the highly conserved transmembrane and cytoplasmic domains 4, 5. Recently, interest in the role of CDH13 in human malignancies has been increasing. Downregulation of CDH13 is frequently observed in many types of cancer, including ovarian carcinoma 6, gastric cancer 7, colorectal cancer 8, bladder transitional cell carcinoma 9, breast cancer 10 and gallbladder cancer^11^, and is generally associated with poor prognosis 12. The overexpression of CDH13 effectively inhibited the proliferation and invasion of tumor cells derived from glioma, cutaneous squamous cell carcinoma and oral squamous cell carcinoma 13-15. Huang et al demonstrated that CDH13 mediated G2 arrest by inducing the expression of cyclin-dependent kinase inhibitor 1 13. However, to date, the role of CDH13 in PC is unknown.

Epithelial-mesenchymal transition (EMT) is a cellular process in which cells lose their epithelial characteristics and acquire mesenchymal features. EMT has been associated with various tumor functions, including the malignant progression, cell migration, metastasis, and resistance to therapy of tumors 16. Numerous studies have indicated that EMT is related to invasiveness, which is defined as a process in which cancer cells spread from the primary tissue to surrounding tissues because the cells lose their cell-cell adhesion ability and gain migratory and invasive capabilities 17-19. While undergoing EMT, cells upregulate the expression of mesenchymal markers (e.g., N-cadherin, Vimentin, and fibronectin) and simultaneously downregulate the expression of epithelial markers (e.g., E-cadherin, and α- and γ-catenin) 20, 21.

In the current study, we explored the expression of CDH13 in PC tissues and cell lines. The effects of CDH13 on cell proliferation, migration and invasion were also investigated *in vitro* and *in vivo.* Moreover, we proposed that CDH13 was involved in PC cell EMT, which was possibly mediated by Wnt/β-catenin signaling.

## Materials and methods

### Immunohistochemistry (IHC) of PC tissue microarray (TMA)

The TMA chip, which contained 78 PC cases including 72 pairs of PC tissues and matched nontumorous pancreatic tissues was obtained from Shanghai Outdo Biotech Co. Ltd. Information on the anti-human CDH13 antibody is shown in Supplementary Table S1. The intensity of CDH13 staining was scored on a scale from 0 (negative) to 3 (strong). Moreover, the percentage of CDH13-positive cells was converted to a scaled score of 0 to 4 (0: 0%; 1: < 25%; 2: 26- 50%; 3: 51- 75%; 4: 76- 100%). The final expression score (ranging from 0 to 12) was determined as the product of the intensity score and the percentage score.

### Cell culture

Human PC cell lines (AsPC-1, BxPC-3, CFPAC-1, and PANC-1) were purchased from the American Type Culture Collection. The cells were cultured in DMEM, IMDM or RPMI 1640 medium containing 10% fetal bovine serum (FBS, Life Technologies, Gaithersburg, MD) and 1% penicillin and streptomycin, maintained in a humidified incubator at 37℃ in 5% CO_2_, and harvested with 0.05% trypsin-0.03% EDTA (Thermo Fisher Scientific, Waltham, MA).

### Overexpression of CDH13 in PC cell lines

Full-length human CDH13 gene transcript variant 1 was subcloned into the lentiviral expression vector pCDH-CMV-MCS (System Biosciences, Mountain View, CA) between the *BamH I* and *Not I* sites. The recombinant lentivirus carrying the CDH13 gene was packaged using the pPACKH1 Lentivector Packaging Kit (System Biosciences, Palo Alto, CA) according to the protocol provided by the manufacturer and concentrated via centrifugation with an Amicon Ultra 100K Centrifugal Filter. BxPC-3 and CFPAC-1 cells were infected with the recombinant lentivirus to generate stable CDH13-overexpressing cells. The overexpression of CDH13 in the transfected cells was confirmed by quantitative real-time PCR (qRT-PCR) and western blotting.

### RNA isolation and qRT-PCR

Total RNA was isolated from the cells by using TRIzol reagent (Life Technologies). cDNA was synthesized using reverse transcription with the PrimeScript RT Reagent Kit (TaKaRa, Kusatsu, Japan). The primer sequences employed in this study are shown in Supplementary Table S2. The qRT-PCR assays were carried out by using FastStart Universal SYBR Green Master (Roche Diagnostics, Mannheim, Germany) in a Fast Real-time PCR 7500 System (Applied Biosystems Life Technologies, Foster City, CA) with the following reaction procedure: 95℃ for 1 min, followed by 40 cycles of 95℃ for 15 sec, and 60℃ for 60 sec. Gene expression was normalized to the expression of GAPDH by the 2^-ΔΔCt^ method^22^. Each experiment was performed in triplicate.

### Protein extraction and western blotting

Protein was extracted using RIPA buffer (Beyotime Institute of Biotechnology, Beijing, China) and quantified using a BCA Protein Assay kit (Thermo Scientific, Rockford, IL). Equal amounts of total proteins were separated and transferred onto PVDF membranes. After blocking with 5% nonfat milk for 1 h, the membranes were incubated with primary antibodies (Supplementary Table S1) overnight at 4℃. After extensive washes, the membranes were incubated with horseradish peroxidase-conjugated IgG (Supplementary Table S1) for 1 h at room temperature. The membranes were detected using a Pierce SuperSignal West Pico chemiluminescent substrate (Thermo Scientific) with a Chemi-Doc XRS system (Bio-Rad, Hercules, CA), and densitometric analyses were processed with ImageJ software.

### Cell proliferation assay

Cells were plated at a density of 3000 cells per well in 96-well plates. Afterward, certain volume (10 μl per well) of the CCK-8 (Dojindo, Tokyo, Japan) reagent was added into certain wells at each monitored time (0, 24, 48, 72 and 96 h). After incubation at 37℃ for 2 h, the absorbance at 450 nm was analyzed with a microplate reader (Tecan, Männedorf, Switzerland).

### Wound healing assay

Cells were plated into 6-well plates at a density of 1×10^5^ cells per well with wound healing culture inserts (ibidi, Martinsried, Germany). Then, the cells were cultured overnight before the inserts were removed. After gently washing the wells with phosphate-buffered saline (PBS), the cells were cultured with the corresponding medium containing 20% FBS; and the wounded areas were documented every 1 h at a live cell station under a microscope (Olympus Corporation, Tokyo, Japan). The cell wound healing rate was evaluated by calculating the percentage of the wound area compared with the area of total cells. Each experiment was performed in triplicate.

### *In vitro* migration and invasion assay

For the transwell migration assay, cells were suspended in serum-free medium and planted in the top chamber of the culture inserts with an 8-μm pore-size membrane (1×10^5^ cells/insert). Medium containing 10% FBS was added into the lower chamber as a chemoattractant. After incubation for 16 h, cells remaining in the top chamber of the inserts were carefully removed. The cells adhering to the bottom of the insert membrane were fixed and stained with a dye solution containing 0.1% crystal violet and 20% methanol. For the transwell invasion assay, the culture inserts were coated with an appropriate amount of Matrigel (BD Biosciences, Franklin Lakes, NJ) mixed with precooled medium at a ratio of 1:7. The subsequent experimental procedure was similar to that of the above-described transwell migration assay.

### RNA sequencing and enrichment analysis

Total RNA from CDH13-overexpressing cells or control cells from three independent experiments was pooled and sent to BGI (Shenzhen, China) for RNA-seq analysis. The RNA-seq libraries were constructed by using pretreated RNAs and the TruSeq Stranded Total RNA Library Prep Kit (Illumina, San Diego, CA) and were sequenced by an Illumina HiSeq X10 Sequencer. Clean reads were mapped to the human genome (hg38) with default parameters by Bowtie2. Then, gene expression was calculated by RNA-seq experiments (RSEM). NOISeq was used to produce biologically meaningful rankings of differentially expressed genes (DEGs), and the significant DEGs were selected according to the following criteria: fold change ≥ 2 or ≤ 0.5 and false discovery rate (FDR)-corrected *P* < 0.05.

Gene Oncology (GO) functional annotation and Kyoto Encyclopedia of Genes and Genomes (KEGG) pathway analysis were performed on the significant DEGs between CDH13-overexpressing cells and control cells using the web-accessible functional annotation tool from the Database for Annotation Visualization and Integrated Discovery (DAVID) bioinformatics resource (http://david.abcc.ncifcrf.gov/; version 6.8). The human genome was selected as the background parameter. GO analysis is a bioinformatics method that is mainly used to annotate genes and categorize them according to their biological processes, cellular components and molecular functions; KEGG is a database that contains various kinds of data from large molecular datasets generated using high-throughput experimental technologies. A *P* value < 0.05 was set as the cut-off criterion.

### Orthotopic and subcutaneous murine model of human PC

To assess the effect of CDH13 on tumor progression, we used 5-week-old female BALB/c nude mice to establish *in vivo* orthotopic or subcutaneous tumor models. In brief, the mice were randomly divided into experimental and control groups. For the orthotopic implantation of cancer cells, 2×10^6^ CDH13-overexpressing CFPAC-1 cells or control cells mixed with an equal volume of Matrigel (BD Biosciences) were injected into the pancreas of the mice. All mice were euthanized on day 45 after inoculation, and the tumor-bearing pancreases were excised and weighed. The hepatic and mesenteric metastases, as well as spleen, colon, and kidney were carefully examined under a dissection microscope.

For the subcutaneous mouse model, 1×10^6^ CDH13-overexpressing CFPAC-1 cells or control cells were injected into the back of the nude mice. Five weeks after implantation, the mice were sacrificed and the tumors were removed. The tumor volume was calculated using the following formula: volume (mm^3^) =4π/3×(width/2)^2^×(length/2).

The animal experiments were approved by the Experimental Animal Care Commission at the Shanghai Cancer Institute.

### Hematoxylin and eosin (H&E) staining

The fixed tissues were embedded in paraffin and then sliced into 5 μm sections. After dewaxing, the sections were stained with hematoxylin (Solarbio, Shanghai, China) and eosin (Sangon, Shanghai, China). The metastatic foci were observed under an Olympus DP73 microscope.

### Statistical analysis

All quantitative data are expressed as the mean ± standard error of the mean (SEM). Statistical analysis was performed using SPSS software version 23.0 (SPSS Inc., Chicago, IL). Before the statistical analysis, all the data were tested for normality. The differences between groups were assessed using Student's *t* test (for normally distributed variables) or the Mann-Whitney U test (for nonnormally distributed variables). The differences between rates were tested by χ^2^ or Fisher exact test, if appropriate. The *P*-values less than 0.05 (*P* < 0.05) was considered statistically significant.

## Results

### CDH13 expression was downregulated in PC specimens and cells

**Figure 1:**
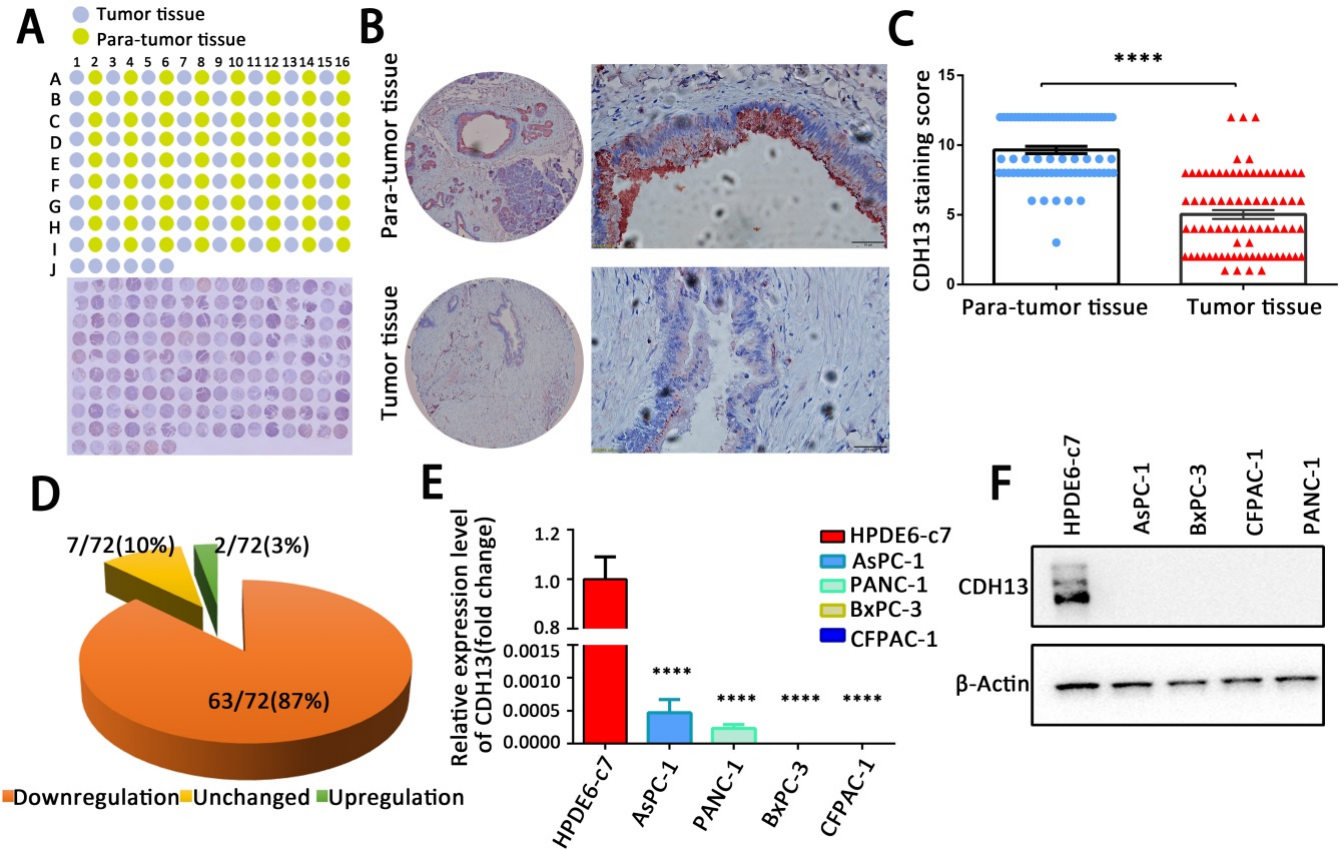
CDH13 was downregulated in human PC tissues and PC cell lines. A, IHC analysis of CDH13 expression in PC tissues compared with that in adjacent para-tumor tissues. B, Representative IHC staining of CDH13 in one PC tissue and its corresponding para-tumor tissue are shown here (original magnification: left panel, 40×; right panel, 400×). C, Comparison of the CDH13 expression score between PC tissues and para-tumor tissues in a tissue microarray containing 72 pairs of tissue samples. D, Changes in CDH13 expression in PC tissues compared with its corresponding para-tumor tissues. E, qRT-PCR analysis of CDH13 mRNA expression in the indicated human PC cell lines and normal pancreatic ductal cell line. The data are based on 3 independent experiments. F, Western blotting of CDH13 protein levels in the indicated PC cell lines and normal pancreatic ductal cell line. The western blot images shown here are representative results of 3 independent experiments. All data are presented as the mean ± SEM. ****P < 0.0001 compared with the controls.

We first examined the expression of CDH13 in a TMA containing 72 pairs of PC tissues and adjacent nontumorous tissues by IHC. As demonstrated in Fig. 1A and B, CDH13 immunoreactivity was shown as granular-like structures in the cytoplasmic membrane and cytoplasm. According to the staining score, CDH13 expression in tumor specimens was lower than that in adjacent nontumorous tissues (*P* < 0.0001, Fig. 1C). Of the 72 paired samples, 63 (87%) samples had lower CDH13 expression in PC tissues than in their corresponding nontumor pancreatic tissues. There were only 7 (10%) and 2 (3.0%) cases that had a similar or higher expression of CDH13 in cancer tissues, respectively (Fig. 1D).

CDH13 expression was also measured in a set of PC cell lines (AsPC-1, BxPC-3, CFPAC-1, and PANC-1) and in one normal human pancreatic ductal cell line (HPDE6-c7) by qRT-PCR and western blotting. The expression of CDH13 in 4 PC cell lines was barely detectable at both the mRNA and protein levels and was significantly lower than that in normal pancreatic ductal cells (Fig. 1E and F). These results indicated that CDH13 is downregulated in PC.

### CDH13 inhibited the proliferation, migration and invasion of PC cells

**Figure 2:**
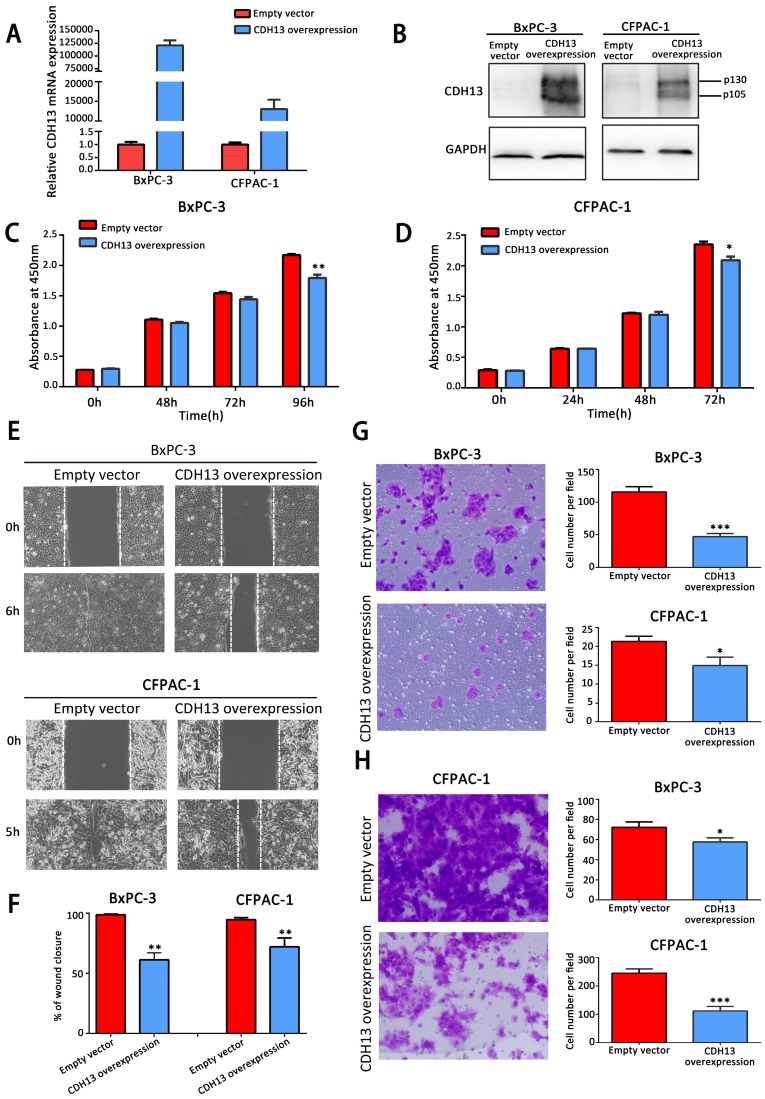
CDH13 overexpression suppressed the proliferation, migration and invasion potential of human PC cells *in vitro.* A-B, qRT-PCR and western blot analysis of CDH13 expression in CDH13 gene-transfected BxPC-3 and CFPAC-1 PC cells and their controls. C-D, The cell proliferation of CDH13-overexpressing cells and control cells was determined via a CCK-8 assay. E, The migratory abilities of BxPC-3 and CFPAC-1 cells after transfection with CDH13-overexpressing cells or control cells were detected by a wound healing assay. F, The gap width was measured using ImageJ software. Three independent experiments were performed to calculate the significance value. G, Cell migratory abilities were also detected in BxPC-3 and CFPAC-1 cell lines by the Transwell migration assay. Representative images of BxPC-3 cells are shown here. H, The invasive potential of CDH13-overexpressing BxPC-3 and CFPAC-1 cells was detected by the Transwell invasion assay. Representative images of CFPAC-1 cells are shown here. The data are presented as the mean ± SEM of 3 independent experiments. *P < 0.05, **P < 0.01, ***P < 0.001 compared with the control cells.

To explore the biological effects of CDH13 on PC, a recombinant lentivirus containing the complete open reading frame of the human CDH13 transcript variant 1 was introduced into BxPC-3 and CFPAC-1 cells to establish stable CDH13-overexpressing PC cells. Cells infected with lentiviruses containing an empty vector were used as the controls. The overexpression of CDH13, which was confirmed by qRT-PCR and western blotting (Fig. 2A and B), significantly inhibited the proliferation of BxPC-3 and CFPAC-1 cells (*P* < 0.05; Fig. 2C and D). The data from the wound healing assay indicated that the overexpression of CDH13 also significantly suppressed the migratory activity of BxPC-3 and CFPAC-1 cells when compared with the corresponding control cells (inhibition rate: 38.8% for BxPC-3 cells and 24.3% for CFPAC-1 cells, both *P* < 0.01) (Fig. 2E). The Transwell migration and invasion assays revealed that CDH13 significantly reduced the migration and invasion of BxPC-3 and CFPAC-1 cells (inhibition rate of migration: 60.0% for BxPC-3 cells and 28.6% for CFPAC-1 cells, both *P* < 0.05; inhibition rate of invasion: 21.35% for BxPC-3 cells and 54.69% for CFPAC-1 cells, both *P* < 0.05, Fig. 2G and H). These data indicated that CDH13 plays a suppressive role in the growth, migration and invasion of human PC cells.

### CDH13 overexpression attenuated tumor progression *in vivo*

**Figure 3:**
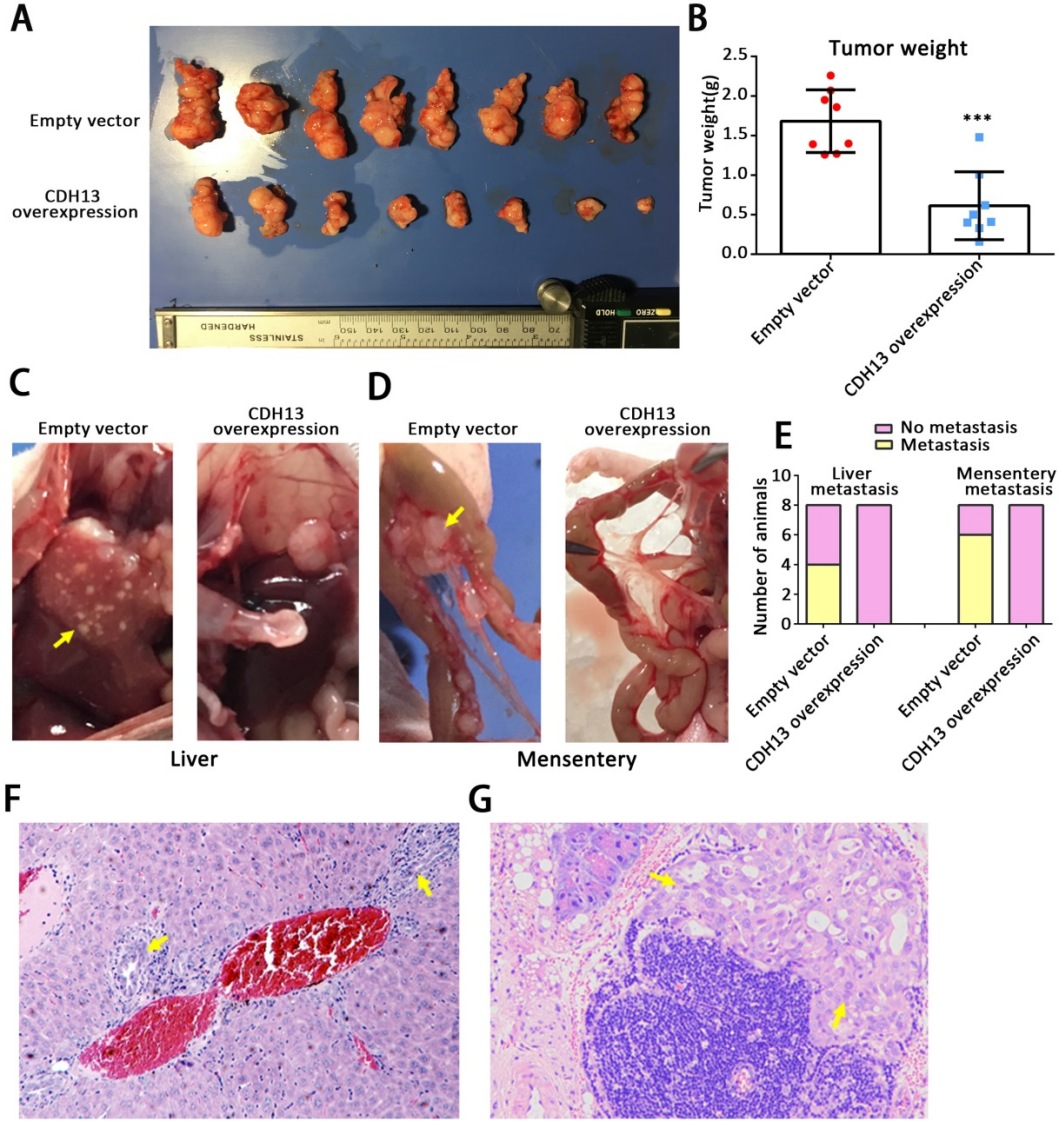
CDH13 overexpression inhibited PC tumor progression *in vivo*. CDH13-overexpressing CFPAC-1 cells were orthotopically injected into the pancreas of nude mice. Cells transfected with the empty vector were used as a control. A, The orthotopic PC tumors removed at the time of euthanasia. B, Comparison of the pancreas weights with orthotopic tumors between the CDH13 overexpression group and control group. C-D, Representative macroscopic images of liver and mesenteric metastases in mice injected with control cells and CDH13-overexpressing CFPAC-1 cells. E, Animals with liver metastasis or mesentery metastasis were counted. F-G, H&E staining indicating the metastatic foci identified in the liver or mesentery of mice with orthotopic PC tumors (original magnification 100×). The data are presented as the mean ± SEM; n = 8 per group. ***P < 0.001 compared with the control group.

To clarify the* in vivo* role of CDH13, we further performed studies in orthotopic tumor and subcutaneous tumor mouse models. After being orthotopically injected with CDH13-overexpressing CFPAC-1 cells or control cells for 6.5 weeks, all mice developed primary tumors in the pancreas. As shown in Fig. 3A and B, the weights of the pancreases containing CDH13-overexpressing tumors were drastically lower than those of the controls (pancreas weight: 0.61 ± 0.15 g* vs* 1.68 ± 0.14 g, *P* < 0.001). Compared with those in the control group, the distant metastases in the CDH13-overexpression group were situated in the liver (0/8 mice *vs*. 4/8 mice, *P* < 0.05, Fig. 3C), mesentery (0/8 mice* vs*. 6/8 mice, *P* < 0.05, Fig. 3D), colon (0/8 mice *vs.* 3/8 mice) and kidney (0/8 mice *vs*. 1/8 mice) (Supplementary Table S3). Histologic analyses further confirmed that the micrometastatic lesions in liver and mesentery (Fig. 3F and G). Similar results were obtained in the subcutaneous tumor models. The CDH13-overexpressing tumors grew significantly slower than the control tumors (Supplementary Fig. S1). Lymph node metastasis occurred in 3/7 mice in the control group, and 0/7 mice in the CDH13-overexpression group (Supplementary Table S4). Although there was no significant difference in lymph node metastasis between the CDH13-overexpression group and the control group, CDH13 overexpression tended to inhibit lymph node metastasis in the subcutaneous tumor models. Collectively, our results indicate that CDH13 inhibits the progression of PC* in vivo*.

### CDH13 attenuated EMT by regulating the canonical Wnt/β-catenin signaling pathway in PC cells

**Figure 4:**
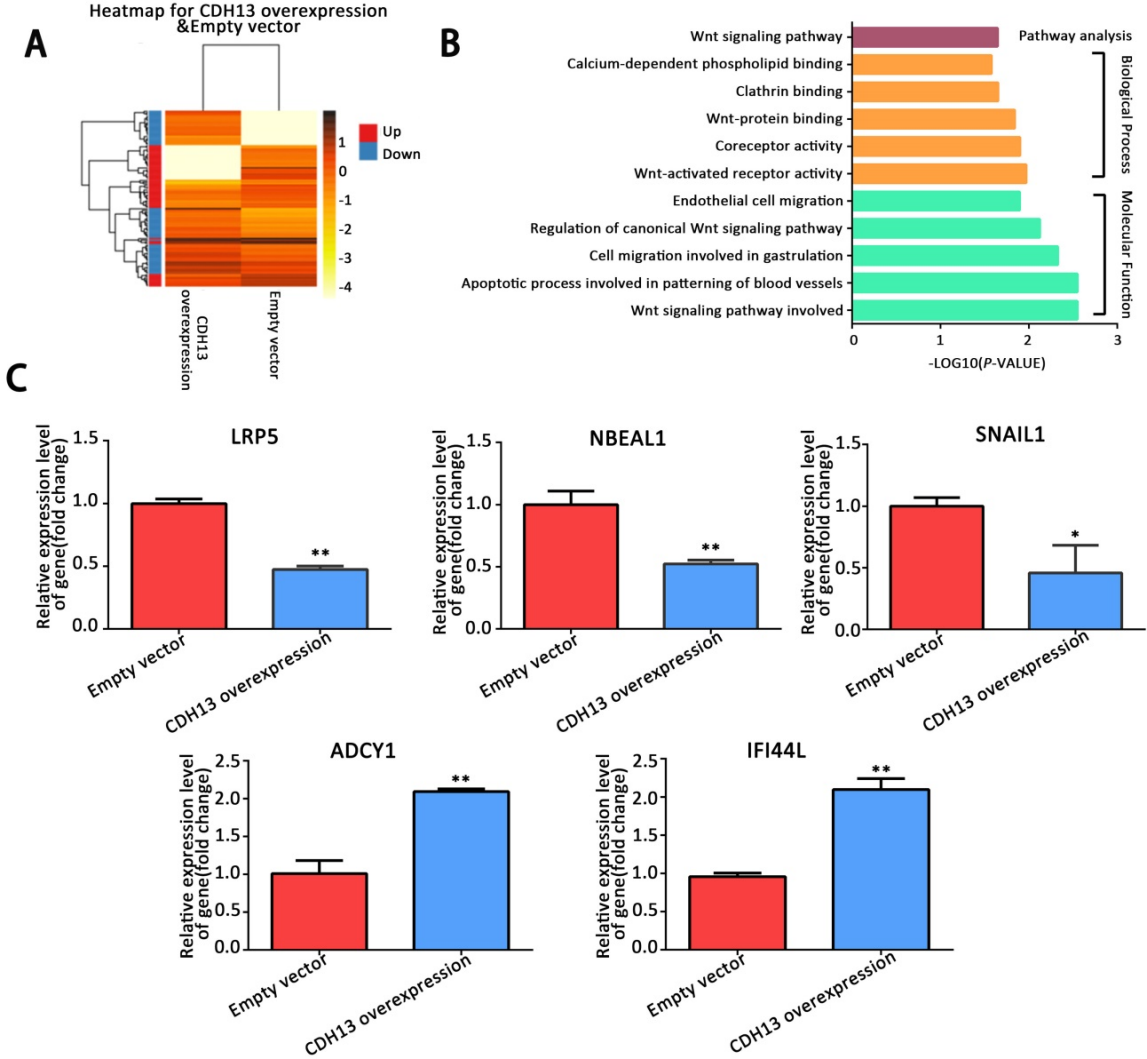
Analysis of differentially expressed genes between CDH13-overexpressing CFPAC-1 cells and control cells by RNA-sequencing. A, Hierarchical clustering illustrated the differentially expressed RNAs in the human PC cell line between CDH13-overexpressing cells and the control cells. Red dots and blue dots represent the upregulated and downregulated RNA, respectively. B, GO and KEGG annotation of differentially expressed genes between CDH13-overexpressing CFPAC-1 cells and control cells. The enriched GO terms (*P* < 0.05) were ranked under different GO subontologies (biological process and molecular function), and only the top 5 terms are shown here. C, Validation of the differentially expressed genes by qRT-PCR. Similar results were seen in 3 independent experiments. The data are shown as the mean ± SEM, **P < 0.01 and *P < 0.05 compared with the control group.

To delineate the mechanisms by which CDH13 inhibited PC progression, we performed a transcriptomics analysis of the mRNA expression profile of CDH13-overexpressing CFPAC-1 cells. A total of 298 genes, including 159 upregulated genes (fold change ≥ 2) and 139 downregulated genes (fold change ≤ 0.5), were found to be differentially expressed in response to CDH13 overexpression (Fig. 4A). GO functional enrichment analysis revealed that the DEGs were mainly enriched in Wnt-activated receptor activity and the Wnt signaling pathway under the biological process and molecular function categories (*P* < 0.05, Fig. 4B). KEGG pathway enrichment analysis also revealed that the DEGs were mainly enriched in the Wnt signaling pathway (*P* < 0.05, Fig. 4B). Some DEGs related to the biological process and molecular function categories were evaluated by qRT-PCR to confirm their mRNA expression changes in CDH13-overexpressing CFPAC-1 cells. Consistent with the results from transcriptomics, qRT-PCR showed that LRP5, SNAIL1 and NBEAL1 were downregulated and that ADCY1 and IFI44L were upregulated in CDH13-overexpressing CFPAC-1 cells (Fig. 4C).

**Figure 5:**
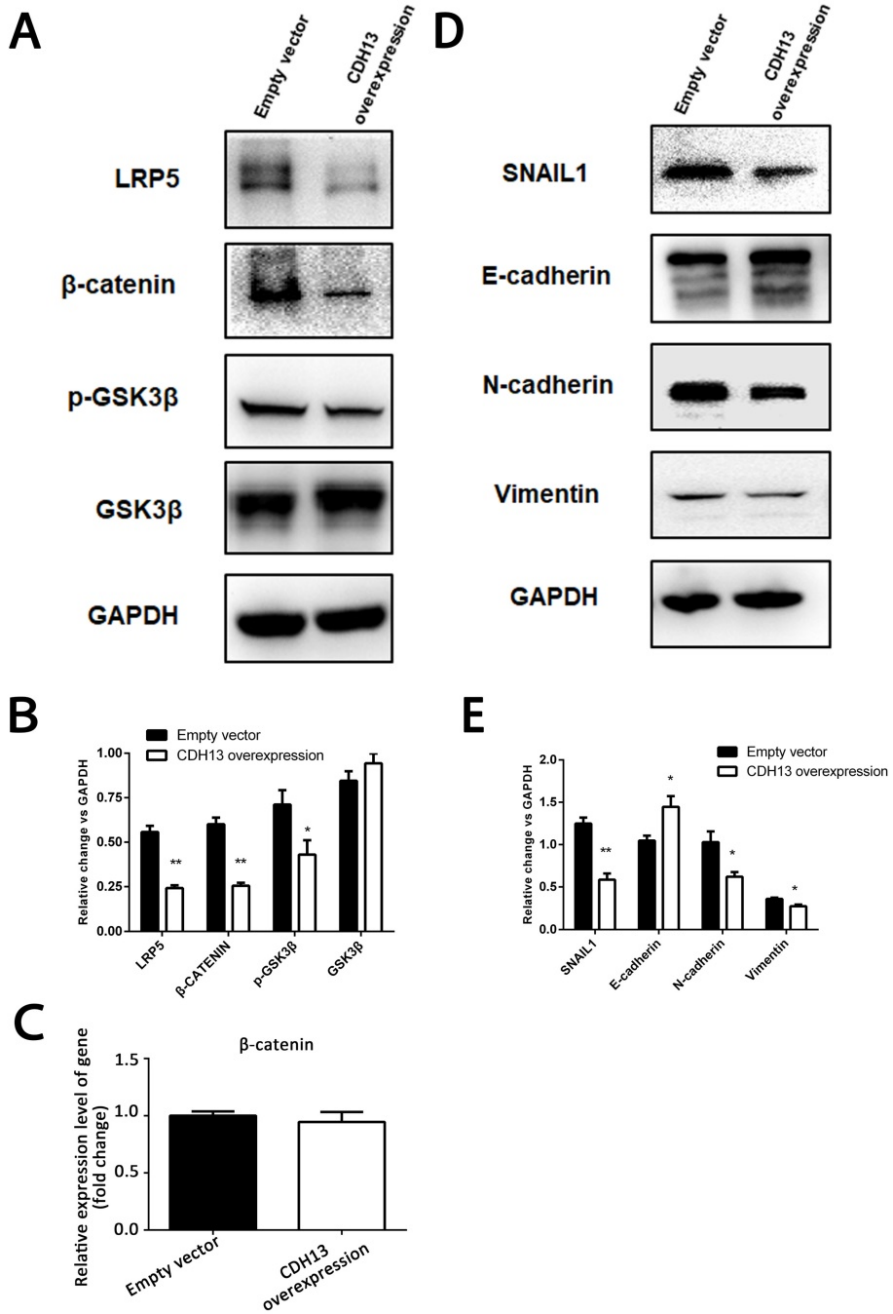
CDH13 overexpression inhibited EMT and the Wnt/β-catenin signaling pathway in PC cells. A-B, Representative images and western blot analysis of LRP5, β-catenin, p-GSK3β and GSK3β between CDH13-overexpressing CFPAC-1 cells and control cells. C, The mRNA expression level of β-catenin was determined by qRT-PCR. D-E, Representative images and western blot analysis of SNAIL1, E-cadherin, N-cadherin and Vimentin between CDH13-overexpressing CFPAC-1 cells and control cells. The data are shown as the mean ± SEM, Similar results were seen in 3 independent experiments. *P < 0.05 and **P < 0.01 compared with the control group.

LRP5/6 are Wnt coreceptors that stabilize β-catenin by increasing the phosphorylation level of GSK3β 23. Since the transcriptomics results demonstrated that LRP5 was downregulated by CDH13 overexpression, we focused our mechanistic studies on the Wnt signaling pathway. As shown in Fig. 5A, the protein levels of LRP5 and β-catenin were evidently decreased after CDH13 overexpression.

However, there was no change at the mRNA expression level of β-catenin (Fig. 5C), suggesting that CDH13 might decrease the protein level of β-catenin by promoting its degradation. Since the proteasomal degradation of β-catenin is mostly controlled by GSK-3β phosphorylation 24, we then examined the phosphorylation state of GSK-3β and found that CDH13 overexpression caused a decreased level of GSK-3β Ser-9 phosphorylation (Fig. 5A). These results suggested an inhibitory effect of CDH13 on the Wnt signaling pathway in human PC cells.

Because our transcriptomic analysis and qRT-PCR assay revealed that SNAIL1, a molecular marker of EMT, was downregulated in CDH13-overexpressing CFPAC-1 cells, we then explored whether the inhibitory effects of CDH13 on cell migration and invasion were related to EMT. As illustrated in Fig. 5D, the expression of E-cadherin was increased with CDH13 overexpression, whereas the expression levels of N-cadherin, SNAIL1 and Vimentin were decreased. Since SNAIL1 is transcriptionally regulated by β-catenin 25, these data suggested that the inhibition of EMT by CDH13 may occur via the suppression of the Wnt/β-catenin signaling pathway.

## Discussion

CDH13 is a newly identified cell adhesion molecule that is located on chromosome 16q24, a region often exhibiting loss of heterozygosity in cancer, including breast, prostate, colon and lung cancer 26-30. The loss or low expression of CDH13 has been observed in a diverse range of cancer types, including cervical cancer, squamous cell carcinoma and melanoma 31-33. However, to date, the expression changes of CDH13 and its biological function in PC have never been investigated. To the best of our knowledge, our current study reported for the first time that CDH13 expression was decreased in PC. Moreover, overexpression of CDH13 inhibited the progression of PC *in vitro* and* in vivo*, supporting its role as a tumor suppressor in PC.

CDH13 is widely expressed in the brain and cardiovascular system but is absent or strongly suppressed in a number of cancer cell lines, such as lung, ovarian, cervical and prostate cancer 34-37. Our observation in PC is in accordance with these reports, demonstrating a lower expression level of CDH13 in all 4 PC cell lines at both the mRNA and protein levels. The IHC results further revealed that CDH13 was downregulated in approximately 87% of PC cases. CDH13 exerted different effects in different kinds of cells. It suppressed proliferation and migration in the majority of cancer cell lines, but it promoted proliferation and migration and had a pro-survival effect in endothelial cells 38.

Most studies have shown that enhanced CDH13 expression inhibited tumor cell growth and metastasis in several *in vitro* and *in vivo* models 32, 39. Our results are consistent with those of previous studies. We showed that the ectopic expression of CDH13 effectively inhibited the proliferation, migration and invasion of PC. Similar results were described in gastric cancer 5, 40, glioma 13 and cutaneous carcinomas 14. To our knowledge, only a few studies have investigated the *in vivo* role of CDH13. Pfaff D et al 39 demonstrated that CDH13 loss was associated with the process of malignant transformation from noninvasive to invasive squamous cell carcinoma (SCC) by a subcutaneous mouse model. Philippova et al 32 reported that the loss of CDH13 increased both the incidence and appearance rate of lung metastases using a murine model of experimental metastasis following the tail vein injection of A431 SCC cells. In our study, mice orthotopically or subcutaneously transplanted with CDH13-overexpressing CFPAC-1 cells developed substantially smaller tumors than the controls, and fewer of them showed metastasis compared with the control mice. However, we failed to knockdown CDH13 expression in PC cells because its expression was barely detectable in all 4 tested PC cell lines. Given that normal human pancreatic ductal cells express a relatively high level of CDH13, further studies may downregulate CDH13 expression in these cells to investigate the effect of CDH13 knockdown on PC initiation.

CDH13 encodes a cell surface glycoprotein belonging to the cadherin family that is responsible for selective cell recognition and adhesion. In human tumors, cell-cell association is often disorganized and thought to be a cause of the unregulated invasion and metastatic behavior of tumor cells 5. Therefore, we speculated that CDH13 might be associated with PC cell migration and invasion. As expected, our results showed that CDH13 overexpression suppressed PC cell migration and invasion; it was associated with the upregulation of E-cadherin expression and the downregulation of Vimentin, N-cadherin and SNAIL expression. E-cadherin, N-cadherin and SNAIL are essential proteins of EMT, which is the most crucial mechanism contributing to cancer invasion and tumor metastasis 41. EMT is characterized by the downregulation of epithelial markers such as E-cadherin and the upregulation of mesenchymal markers, including N-cadherin, SNAIL1 and Vimentin 42. Lin et al revealed that CDH13 might play an important role in gastric cancer metastasis by positively regulating E-cadherin expression and negatively regulating Vimentin expression 5. Similarly, we demonstrated that CDH13 overexpression inhibited the migration and invasion of PC cells possibly by regulating the expression of EMT-related proteins.

Wnt/β-catenin signaling plays a pivotal role in regulating cell proliferation, migration, and invasion 43. In canonical Wnt/β-catenin signaling, Wnt ligands bind to the dual receptor complex comprised of frizzled and LRP5/6. This leads to the inactivation of the destruction complex Axin/APC/GSK-3β, and the critical mediator β-catenin is relieved from its constitutive proteasomal degradation 41. β-catenin subsequently accumulates in the cytoplasm and translocates into the nucleus, where it associates with transcription factors to regulate downstream target genes 44. Our data showed that CDH13 overexpression decreased the levels of LRP5. In the absence of the Wnt signal, the phosphorylation of GSK3β at serine 9 was inhibited, which led to the degradation of β-catenin and subsequent reduced transcription of its target gene SNAIL1, which is responsible for EMT and cancer progression. Tang et al reported that the suppression of E-cadherin through its transcriptional repressor SNAIL1 was a determining factor for EMT 45. This pattern was observed in the present study, in which CDH13 overexpression enhanced E-cadherin and suppressed SNAIL1. Because SNAIL1 is one of the β-catenin regulated genes, and Wnt/β-catenin signaling is associated with cell invasion and EMT 25, 46, our data thus suggested that the inhibitory effects of CDH13 on PC invasion and EMT may be mediated via the Wnt/β-catenin signaling pathway.

Overall, our data demonstrated that CDH13 was significantly decreased in PC tissues and cells. Furthermore, overexpression of CDH13 exerted *in vivo* and *in vitro* antiproliferative, anti-EMT and antimetastatic activities against human PC (summarized in Supplementary Table S5). These activities could have been mediated through the inhibition of the Wnt/β-catenin signaling pathway. These results provide a new perspective of CDH13 as an inhibitor of PC progression. Nevertheless, some limitations are present in this study. For example, functional experiments were not performed to verify that CDH13 inhibits the metastasis of PC, as well as the EMT of PC cells, by regulating the β-catenin signaling pathway. Further analyses are warranted to characterize the signaling cascade by which CDH13 regulates the GSK-3β/β-catenin pathway in PC.

## Supplementary Material

Supplementary figure and tables.

## Figures and Tables

**Figure 6:**
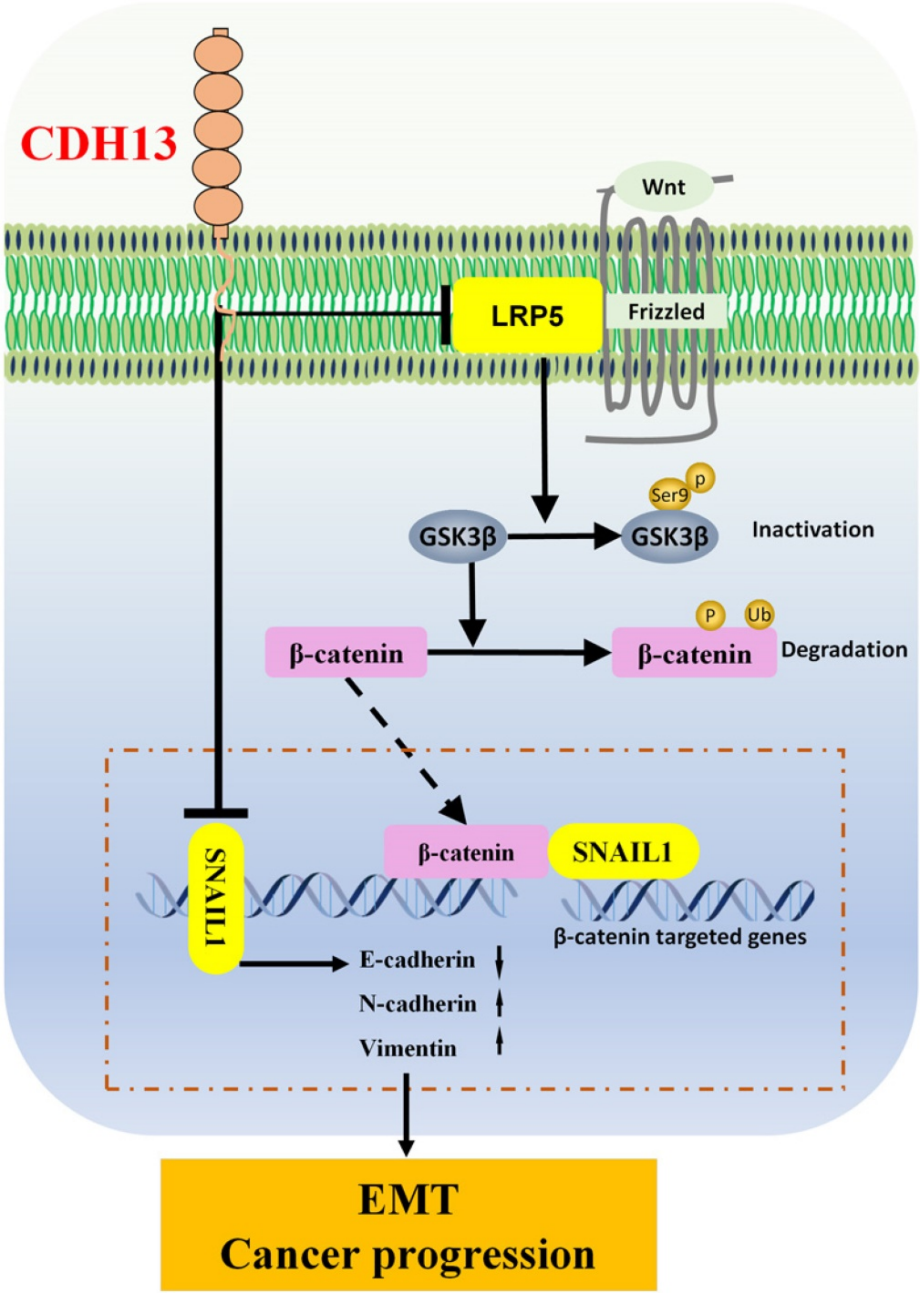
Schematic diagram of the signaling pathways involved in the CDH13-induced inhibitory effect on human PC cells. CDH13 inhibits the Wnt signal by downregulating the expression of LRP5, which is one of the Wnt receptors. In the absence of Wnt signals, the phosphorylation of GSK3β at serine 9 is inhibited, which leads to the degradation of β-catenin and subsequent decreased transcription of its target gene SNAIL1. Consequently, the EMT and progression of pancreatic cancer cells are inhibited.

## Abbreviations

CDH13: cadherin 13
PC: pancreatic cancer
EMT: epithelial-mesenchymal transition
IHC: Immunohistochemistry
qRT-PCR: quantitative real-time PCR
TMA: tissue microarray chip
H&E: Hematoxylin and eosin staining
