# Supplementary material for: Cadherin 13 Inhibits Pancreatic Cancer Progression and Epithelial-mesenchymal Transition by Wnt/β-Catenin Signaling
Source: J Cancer. 2020 Feb 3;11(8):2101–12. doi: 10.7150/jca.37762 (PMC7052920; doi:10.7150/jca.37762)
Supplement: Supplementary file 1 — Supplementary figure and tables. [file jcav11p2101s1.pdf]

Supplementary Materials

Supplementary Tables

Supplemental Table S1: The Detailed Information on Antibodies Used for IHC and Western Blot

Antibody	Vendor	Source	Working dilution
anti-CDH13	R&D systems	goat	IHC&WB: 1:1000
anti-E-cadherin	Signalway Antibody	rabbit	WB: 1:1000
anti-β-catenin	Signalway Antibody	rabbit	WB: 1:1000
anti-Vimentin	Santa Cruz Biotechnology	rabbit	WB: 1:200
anti-N-cadherin	Santa Cruz Biotechnology	rabbit	WB: 1:200
anti-SNAIL1	Cell Signaling Technology	mouse	WB: 1:500
anti-GSK3β	Cell Signaling Technology	rabbit	WB: 1:1000
anti-phos-GSK3β	Cell Signaling Technology	rabbit	WB: 1:1000
anti-GAPDH	Cell Signaling Technology	Rabbit	WB: 1:5000
Donkey Anti-Goat IgG(H+L)	Proteintech	Donkey	WB: 1:2000
Goat Anti-Rabbit IgG(H+L)	Proteintech	Goat	WB: 1:2000
Goat Anti-Mouse IgG(H+L)	Proteintech	Goat	WB: 1:2000

Supplemental Table S2: Primer sequences of quantitative real-time PCR

Gene	Sequences 5'-3'
CDH13	TCCCTGCAGCATCAAACCAT
	ACAAATGGGGACTCACGGTC
LRP5	CTGTACCCGCCGATCCTGA
	GGCGCCATTCCTCGAATGAT
NBEAL1	CCAGACAGTGGGAAAACCGA
	TCCCTGAAACACCTTGCAGTT
SNAIL1	TAGCGAGTGGTTCTTCTGCG
	AGGGCTGCTGGAAGGTAAAC
IFI44L	CCTCTTCTAACAAACCCATGCT
	AGCTTTCACAGCTAGTAAGAGGA
ADCY1	GAGGGGACAAGGAAGGTGC
	CAAAAGGAGCTGCCAAACCC
β-catenin	ACGGAGGAAGGTCTGAGGAG
	GAGTAGCCATTGTCCACGCT
GAPDH	GACCCCTTCATTGACCTCAACTAC
	TGGTGGTGCAGGATGCATTGCTGA

Supplemental Table S3: The effect of CDH13 in orthotopic mouse model of pancreatic cancer

	Empty vector	CDH13 overexpression	
	MEAN ± SEM	MEAN ± SEM	*P*-value
Tumor weight(g)	1.6830 ± 0.1402	0.6138 ± 0.1520	0.000*
Liver weight(g)	1.3360 ± 0.0285	1.1730 ± 0.0464	0.009*
Liver metastasis(case)	4/8	0/8	0.038*
Colon metastasis(case)	3/8	0/8	0.055
Mesentery metastasis(case)	6/8	0/8	0.003*
Kidney metastasis(case)	1/8	0/8	0.500

* $P < 0.05$

Supplemental Table S4: The effect of CDH13 in subcutaneous mouse model of pancreatic cancer

	Empty vector	CDH13 overexpression	
	MEAN ± SEM	MEAN ± SEM	*P*-value
Tumor weight(g)	0.7000 ± 0.0445	0.4871 ± 0.0861	0.049*
Lymph node metastasis(case)	3/7	0/7	0.096

* $P < 0.05$

Supplemental Table S5: Significant findings in this manuscript

Significant findings	*P* values
CDH13 expression was downregulated in PC specimens and cells	
Downregulation of CDH13 expression (tumor tissues *vs* para-tumor normal tissues)	$P < 0.001$
Downregulation of CDH13 expression (PC cells *vs* normal pancreatic ductal cells)	$P < 0.001$
CDH13 overexpression inhibited the cell viability, migration and invasion of PC cells *in vitro*	
Decreased cell viability (CDH13-overexpressing cells *vs* control cells)	$P < 0.05$
Decreased cell migration (CDH13-overexpressing cells *vs* control cells)	$P < 0.05$
Decreased cell invasion (CDH13-overexpressing cells *vs* control cells)	$P < 0.05$
CDH13 inhibited tumor growth and metastasis *in vivo*	
Decreased tumor weight (CDH13 overexpression group *vs* empty vector group)	$P < 0.05$
Decreased tumor metastasis (CDH13 overexpression group *vs* empty vector group)	$P < 0.05$
CDH13 overexpression exerted anti-EMT effect in CFPAC-1 cells	
Upregulation of E-cadherin expression (CDH13-overexpressing cells *vs* control cells)	$P < 0.05$
Downregulation of N-cadherin, Vimentin, and SNAIL1 expression (CDH13-overexpressing cells *vs* control cells)	All $P < 0.05$
CDH13 overexpression inhibited the activation of the Wnt/β-catenin signaling pathway	
Downregulation of LRP5, β-catenin, and p-GSK3β expression (CDH13-overexpressing cells *vs* control cells)	All $P < 0.05$

Supplementary Figure

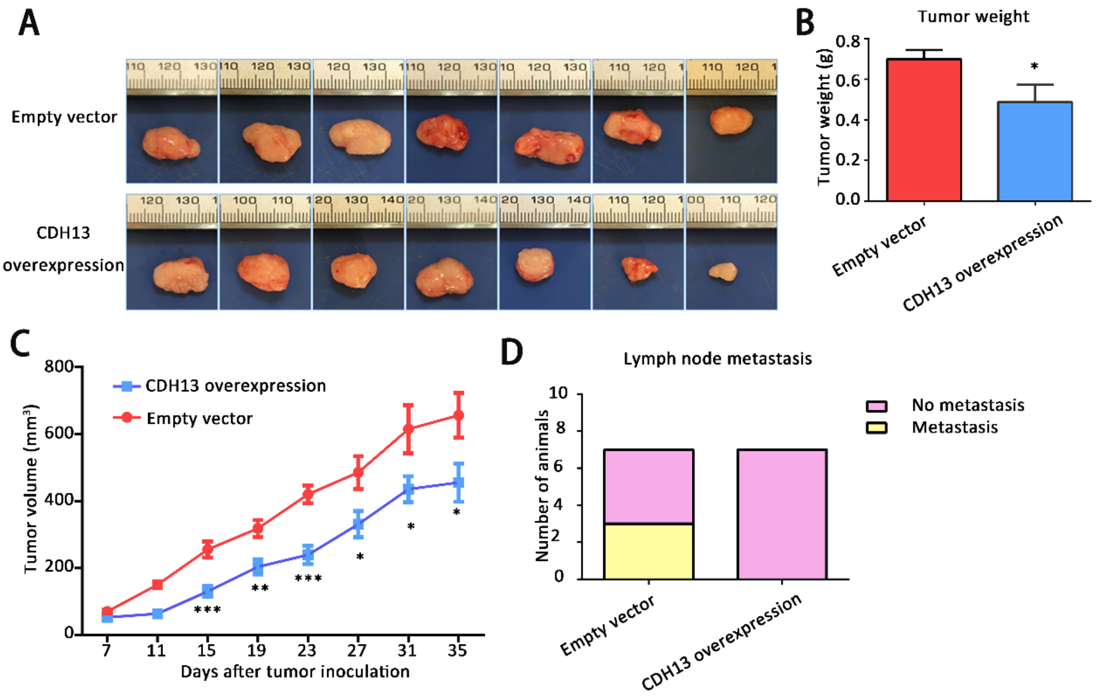

Supplementary Figure S1. CDH13 overexpression inhibited PC progression in subcutaneous tumor model. CDH13-overexpressing CFPAC-1 cells were subcutaneously injected into the nude mice. Cells transfected with the empty vector were used as a control. A, The subcutaneous PC tumors dissected at the time of euthanasia. B, The comparison of the weights between the CDH13-overexpressing tumors and controls. C, Growth curve based on the volumes of CDH13-overexpressing tumors and controls. D, The animals with lymph node metastasis in each group were counted. Data are presented as the mean ± SEM; n = 7 per group. * $P < 0.05$, ** $P < 0.01$ and *** $P < 0.001$, compared with the control group.
